# Intelligence and all-cause mortality in the 6-Day Sample of the Scottish Mental Survey 1947 and their siblings: testing the contribution of family background

**DOI:** 10.1093/ije/dyx168

**Published:** 2017-08-21

**Authors:** Matthew H Iveson, Iva Čukić, Geoff Der, G David Batty, Ian J Deary

**Affiliations:** 1 The Centre for Cognitive Ageing and Cognitive Epidemiology, The University of Edinburgh, Edinburgh, UK; 2 The Administrative Data Research Centre Scotland, Edinburgh, UK; 3 MRC Social and Public Health Sciences Unit, University of Glasgow, Glasgow, UK; 4 Department of Epidemiology and Public Health, University College, London, UK

**Keywords:** mortality, intelligence, socio-economic status

## Abstract

**Background:**

Higher early-life intelligence is associated with a reduced risk of mortality in adulthood, though this association is apparently hardly attenuated when accounting for early-life socio-economic status (SES). However, the use of proxy measures of SES means that residual confounding may underestimate this attenuation. In the present study, the potential confounding effect of early-life SES was instead accounted for by examining the intelligence–mortality association within families.

**Methods:**

The association between early-life intelligence and mortality in adulthood was assessed in 727 members of the 6-Day Sample of the Scottish Mental Survey 1947 and, for the first time, 1580 of their younger siblings. These individuals were born between 1936 and 1958, and were followed up into later life, with deaths recorded up to 2015. Cox regression was used to estimate the relative risk of mortality associated with higher IQ scores after adjusting for shared family factors.

**Results:**

A standard-deviation advantage in IQ score was associated with a significantly reduced mortality risk [hazard ratio = 0.76, *p* < 0.001, 95% confidence interval (CI) (0.68–0.84)]. This reduction in hazard was only slightly attenuated by adjusting for sex and shared family factors [hazard ratio = 0.79, *p* = 0.002, 95% CI (0.68–0.92)].

**Conclusions:**

Although somewhat conservative, adjusting for all variance shared by a family avoids any potential residual confounding of the intelligence–mortality association arising from the use of proxy measures of early-life SES. The present study demonstrates that the longevity associated with higher early-life intelligence cannot be explained by early-life SES or within-family factors.

**Key Messages**

- A standard-deviation increase in IQ score was associated with a 24% decrease in mortality risk.
- After adjusting for all shared family factors, a standard-deviation increase in IQ score was associated with a 21% decrease in mortality risk.
- Early-life socio-economic circumstances are not sufficient to explain the intelligence–mortality association.

## Introduction

Identifying predictors of mortality and chronic disease has been the goal of many epidemiological studies from across various countries and populations. In cognitive epidemiology, intelligence (general cognitive function) as measured using psychometric tests in middle- and older-aged populations has emerged as a predictor of longevity, with higher intelligence test scores associated with reduced mortality risk.^1–5^ More recently, pre-morbid measures of cognitive ability in cohorts of children and young people have been shown to be related to mortality risk up to seven decades later.^6–10^

A key consideration in interpreting these results is understanding the role of early-life socio-economic status (SES). Early-life SES may be a key confounder in the cognition–mortality relation owing to its association with both childhood^11^ and adult intelligence,^12^ and its link to later-life mortality and health.^7^^,^^13–16^ However, the causal relationship between early-life SES and intelligence, and thus the nature of the resulting association with later-life mortality, is unclear.^17^^,^^18^ Researchers have typically attempted to account for early-life SES by including it as a predictor in multivariable models, and many have observed that doing so does not attenuate the contribution of early-life intelligence to mortality risk.^10^

However, attempts to account for any contribution of early-life SES to the intelligence–mortality association are limited by the way in which SES is operationalized. Given the breadth and complexity of SES, it is difficult to fully characterize early-life social circumstances. Researchers commonly include proxy measures for early-life SES, such as parental occupation,^19^ parental income^7^ or participant’s own education.^15^ This has also resulted in a lack of consistency between cohort studies in terms of the SES measure used.^20^ Given these shortcomings, it is likely that not all of the variance associated with early-life SES has been fully captured or accounted for in studies examining the association between intelligence and mortality. Residual confounding, resulting from measurement error in early-life SES, may therefore be driving previously observed associations to some extent, even after adjusting for apparent early-life SES.

In a study of the link between intelligence in youth and later income inequality, Murray^21^ attempted to tackle this SES-assessment problem by examining associations within families. Having matched individuals to their nearest siblings, Murray then examined whether within-sibling-pair intelligence differences could predict income differences within pairs. Examining the contribution of intelligence within families circumvented the need to obtain a range of SES measures, as it removed the variance accounted for by the shared family environment (e.g. parental income, occupation and education). Examining outcomes within families therefore allows researchers to account for early-life SES without the need to operationalize and measure it.

The present study adopts a within-family method in order to examine the association between early-life intelligence and mortality while accounting for early-life SES. Families were established by linking members of the 6-Day Sample of the Scottish Mental Survey 1947 with their younger siblings. The 6-Day Sample is a group of individuals (*N* = 1208) representative of the whole Scottish population born in 1936 and whose cognitive ability was tested at age 11 years old.^22^^,^^23^ Previous work with this sample has shown early-life intelligence to be a significant predictor of mortality from up to 67 years old, even after early-life SES (interviewer-rated parental intelligence and personality, household cleanliness, etc.) had been accounted for.^24^ Importantly, the younger siblings of these individuals were tested on the same IQ-type test when they reached 11 years old, and were recently linked to records of mortality. These siblings shared similar early-life circumstances and upbringings to their 6-Day Sample probands (e.g. parental SES and household size), but may differ in their cognitive ability and longevity. By examining the intelligence–mortality association within families, each consisting of a 6-Day Sample member and their siblings, it is possible, more comprehensively than previously, to partition out the potential confounding effect of early-life SES.

## Methods

### Study sample

On 4 June 1947, almost all children born in 1936 and attending school in Scotland sat the Moray House Test No. 12 test of intelligence.^22^ This cohort of 70 805 individuals, the Scottish Mental Survey 1947, comprised 88% of the Scottish population born in 1936^25^ and 94% of the available school population at the time.^26^ A subsample of this 1936 birth cohort, the 6-Day Sample (*n* = 1208; 618 females), was created by selecting individuals born in Scotland on the first day of every even-numbered month in 1936, whether or not they completed the Moray House Test of intelligence.^22^ The mean intelligence and geographical distribution of the 6-Day Sample has been shown to be similar to the full Scottish Mental Survey 1947 cohort.^27^ Members of the 6-Day Sample were given a second, individually administered test of intelligence in 1947—the Terman-Merrill revision of the Binet Test^22^^,28^—and were subsequently resurveyed about every year up to the age of 27 years to collect information on education, family life, home environment, leisure activities, health and early-adulthood occupation.^23^^,^^25^

Younger siblings (*n* = 1655; 798 females) of the 6-Day Sample members were tested for intelligence using the same Terman-Merrill Binet Test as they approached age 11 years, and were also resurveyed into early adulthood. Of the whole 6-Day Sample, 748 had siblings included in the follow-up surveys. The Sibling Sample, born between 1937 and 1958, ranged from first siblings (*n* = 748) to tenth siblings (*n* = 3), and siblings were on average 6.13 years younger than their 6-Day Sample probands [standard deviation (SD) = 3.96, Min. = 0.39 years, Max. = 22.36 years]. Fourteen of the Sibling Sample were twins, though none was twinned with their 6-Day Sample proband.

Combining the two cohorts resulted in a sample of 2863 individuals. Of these, 79 individuals (6-Day Sample: *N* = 4, Sibling Sample: *N* = 75) for whom follow-up data were not available were removed from any further analysis. As part of previous work, formal comparisons have shown that individuals lost to follow-up demonstrate higher childhood IQ scores (a difference of 4.7 IQ points), higher levels of schooling and higher SES relative to those retained in the 6-Day Sample.^29^ In order to ensure that family-related factors could be shared, the present study focused on multiple-child families. Excluding those 6-Day Sample members from single-child families (*N* = 460) or those 6-Day Sample members whose siblings were removed due to missing data (*N* = 17) resulted in a total sample of 2307 individuals (6-Day Sample: *N* = 727; Sibling Sample: *N* = 1580) from 728 families (mean family size = 3.13, SD = 1.57).

### Assessments

#### Intelligence

For both the 6-Day Sample and their siblings, intelligence was measured using the Terman-Merill test, Form L^30^—an adapted version of the Binet-Simon test of intelligence. This test included 129 items of both verbal and non-verbal reasoning. Raw correct scores were converted into standardized IQ-type scores (*M* = 100, SD = 15). For the 6-Day Sample, this test was administered in 1947, following their completion of the Scottish Mental Survey 1947. Siblings, on the other hand, completed the test at age 11 years, between 1948 and 1969.

#### Mortality

Vital status and date of death were obtained for each of the 6-Day Sample members and their siblings. This was achieved by linking both the 6-Day Sample and their siblings to their respective administrative records held by the National Records of Scotland (NRS). This linkage was approved by the Scotland-A Research Ethics Committee (Ref: 12/SS/0024), the National Services Scotland NHS Privacy Advisory Committee and the Confidentiality Advisory Group of the Health Research Authority. Approval covered linkage without consent, up to November 2015, under section 251 if the NHS Act 2006. The NRS used automated and manual tracing methods to link identifiable information (date of birth, surname, forename and National Health Service number) for each individual with their respective National Health Service Central Register records.

Individuals were censored at the end of mortality surveillance (30 November 2015 for most individuals). Survival time was calculated as the number of days between the date of birth and either the date of death or censoring date as appropriate. Individuals who had emigrated after completing the intelligence test but before the end of the surveillance period were retained in the sample, but were censored at the start of the month in which they embarked.

### Statistical analyses

Survival analyses were conducted using Cox proportional hazards regression. Hazard ratios were calculated for each predictor included in the model to indicate the proportionate change in mortality risk for a unit change in the predictor. In the first set of analyses, individual univariable regression models were created to predict survival using either standardized (*z*-transformed) IQ scores, sex or family size. Each model additionally included a random effect of family to account for the fact that observations within each family are correlated. All of the predictors conformed to the proportional hazards assumption (all *p*s > 0.28). In the second set of analyses, the effect of standardized IQ scores, sex and family size were assessed after adjusting for the effects of all other predictors, including the random effect of family. In the third set of analyses, the mutually adjusted effects of standardized IQ scores and sex were assessed in a fixed-effects model that was estimated by stratifying the analysis on family. This allows a different baseline hazard for each family, and allows the effects of shared family factors to be absorbed without having to be estimated or even measured. Thus, the contribution of standardized IQ scores to survival is assessed independently of all shared family factors. However, the stratified model cannot estimate the effect of family size, as family size is constant within families. In all cases, missing values were deleted in a listwise manner.

Analyses were conducted in R (v3.3.1)^31^ using the ‘psych’ package (v1.6.6).^32^ Cox regression analyses were conducted using the ‘survival’ (v2.40–1)^33^ and ‘coxme’ (v2.2–5)^34^ packages.

## Results

Table 1 shows the descriptive characteristics of the 6-Day Sample and Sibling Sample members. Members of the 6-Day Sample exhibited significantly higher IQ scores than the members of the Sibling Sample, though this only equated to a mean difference of 1.97 IQ points (Table 1). This difference was likely due to the combination of family size effects^22^^,^^27^ and the way in which the comparison was weighted: as all younger siblings were retained in the comparison, larger families contributed more IQ scores to the sibling IQ distribution, thus lowering the mean IQ score for siblings. Indeed, a comparison between 6-Day Sample members and their nearest siblings by age demonstrated no significant difference in IQ scores (*p* = 0.368). Similarly, 6-Day Sample members exhibited significantly longer survival times, both for those alive at the censor date and for those who had died, than members of the Sibling Sample (Table 1). This reflects the fact that all individuals included in the Sibling Sample were younger than those in the 6-Day Sample, and therefore did not have the opportunity to accrue the same length of exposure period.

**Table 1.** Descriptive characteristics of the 6-Day Sample members and their younger siblings, and comparisons between groups

	6-Day Sample (*N* = 727)		Sibling Sample (*N* = 1580)	
	Mean	SD	Mean	SD
Sex (*N* male/female)	363/364		819/761	
IQ score*	100.28	19.05	98.31	17.60
Mortality status (*N* alive/dead)	437/290		1144/436	
Time to death (years, from birth)**	63.99	11.65	57.93	14.35
Time to censor (years, from birth)**	79.43	0.28	72.93	4.12

Missing values were deleted listwise in each of the variable estimates. Time to death is calculated only for those who have died before the censor date; time to censor is calculated only for those still alive at the censor date. *t-test conducted between the 6-Day Sample and Sibling Sample, *p* = 0.019; **p < 0.001.

Table 2 shows the descriptive statistics of the whole sample (6-Day Sample individuals and Sibling Sample individuals combined) according to mortality status. Those individuals who were still alive at the censor date demonstrated significantly higher IQ scores than those who had died.

**Table 2.** Descriptive characteristics of the whole sample (*N* = 2307) of 6-Day Sample members and their younger siblings according to mortality status

	Alive (*N* = 1581)		Dead (*N* = 726)	
	Mean	SD	Mean	SD
Sex (*N* male/female)	735/846		447/279	
IQ score*	100.73	18.20	95.09	17.28
Family size (people)	5.36	2.75	5.40	2.63
Survival time (years, from birth)*	74.59	5.10	60.34	13.66

Missing values were deleted listwise in each of the variable estimates. Survival time for those dead individuals represents the time until death; survival time for those alive represents the time until the censor date. *t-test conducted between those alive and dead, *p* < 0.001.

Table 3 shows three sets of Cox regression analyses: first, the hazard ratios of standardized IQ score, sex and family size individually, with family entered as a random effect in each univariable model; second, the mutually adjusted hazard ratios from a multivariable mixed-effects model of standardized IQ score, sex and family size with family entered as a random effect; and third, the mutually adjusted hazard ratios from a multivariable fixed-effects model of standardized IQ score and sex stratified by family.

**Figure 1:**
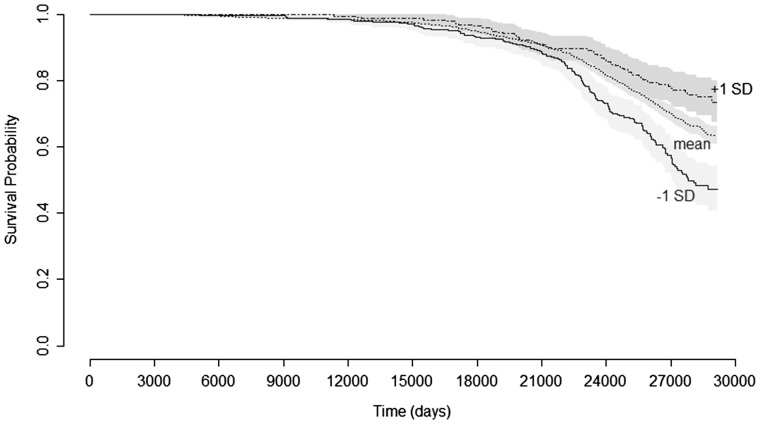
Kaplan–Meier survival curves for members of the full sample based on IQ scores. Lines show survival probability for those with mean IQ scores and for those with IQ scores 1 standard deviation above or below the mean. Shaded areas represent 95% confidence intervals.

Figure 1 shows the change in survival probability for those with a standard-deviation advantage or disadvantage in IQ score. In the univariable model, after accounting for the correlation between family members, a standard-deviation advantage in IQ score was significantly associated with a 24% reduction in mortality risk (Table 3). Once the effects of sex and family size were accounted for, a standard-deviation advantage in IQ score was associated with a 27% decrease in mortality risk. In the stratified multivariable model, in which a different baseline mortality risk was specified for each family, the hazard ratio for standardized IQ scores was somewhat attenuated, but there remained a significant association between a standard-deviation advantage in IQ scores and a 21% reduction in mortality risk.

**Table 3.** Hazard ratios (HRs) showing the mortality risk associated with a 1 standard-deviation increase in IQ score, with being female and with a one person increase in family size. Shown are the HRs including the random effect of family (in the univariable models), adjusted for other predictors and including the random effect of family (in the multivariable models), and adjusted for other predictors and the stratifying effect of family (in the stratified multivariable model; *N* = 2228)

	Univariable			Multivariable			Stratified multivariable		
	HR	95% CI	*p*	HR	95% CI	*p*	HR	95% CI	*p*
Standardized IQ score	0.76	0.68–0.84	<0.001	0.73	0.64–0.82	<0.001	0.79	0.68–0.92	0.002
Sex (Female)	0.57	0.41–0.72	<0.001	0.53	0.37–0.68	<0.001	0.47	0.38–0.58	<0.001
Family size	1.03	1.00–1.06	0.043	0.99	0.96–1.03	0.760	–	–	–

Mortality risk was significantly lower for females in the univariable model. The hazard ratio associated with being female was not attenuated when adjusted for standardized IQ score and family size, nor by stratifying the analyses by family. In the stratified multivariable model, being female was associated with a 53% lower mortality risk. Family size, on the other hand, was associated with a higher risk of mortality in the univariable model, with a 3% increase in mortality risk for a one-member increase in family size. This association was attenuated once the effects of standardized IQ score and sex were adjusted for.

The association between IQ scores and mortality remained relatively consistent across three further analyses (see Supplementary Material, available as Supplementary Data at *IJE* online). First, including single-child families (*N* = 2784 individuals) in the analyses demonstrated a 22% reduction in mortality risk with a standard-deviation advantage in IQ score after adjusting for shared family factors. Second, including only those multiple-child families in which siblings were born within 7 years of their 6-Day Sample probands (*N* = 1713 individuals) demonstrated a 23% reduction in mortality risk with a standard-deviation advantage in IQ score after adjusting for shared family factors. Third, repeating the survival analyses instead adjusting for an explicit proxy measure of SES, father’s occupational social class, demonstrated a 26% reduction in mortality risk with a standard-deviation advantage in IQ score.

## Discussion

The present study uses, for the first time to our knowledge in cognitive function-survival analyses, proband and sibling data. These valuable sibling data accompany the 6-Day Sample of the Scottish Mental Survey 1947, and were used to examine the association between early-life intelligence and mortality independently from family-related early-life SES. The role of early-life SES in predicting mortality has been well established,^14^^,^^16^ and previous studies of early-life intelligence have tended to take and account for some measure of SES (e.g. parental occupation) in subsequent survival analyses to try to test for possible confounding by family background. However, such attempts are limited by the lack of consensus on which measure of SES to account for^20^ and by the residual confounding effect resulting from using select or few measures of SES. The present study tackles these limitations by examining the association between intelligence and mortality within families of the 6-Day Sample and their siblings, thus accounting for shared family factors without the need to operationalize SES. After accounting for family-related SES in this fashion, there remained a significant and only slightly attenuated association between early-life intelligence and longevity. In the most conservative of the analyses, we observed a 21% decrease in mortality risk with each standard-deviation advantage in IQ score. This observation is consistent with previous reports of SES-adjusted hazard ratios in the Scottish Mental Survey 1947 (20% decrease in mortality risk)^26^ and in the 6-Day Sample more specifically (from 16% to 26% decrease in mortality risk).^24^ The persistence of the intelligence–mortality association after taking into account early-life SES is also consistent with a recent meta-analysis of nine prospective cohort studies,^10^ in which adjusting for various measures of SES did not attenuate the reduction in mortality risk associated with higher intelligence test scores (23% decrease in mortality risk after adjustment). Also consistent with previous work was the observed associations between male sex and increased mortality risk^24^ and between larger family size and increased mortality risk.^35^

The somewhat attenuating effect of adjusting for family-related SES, although small, is broadly consistent with previous work using discrete proxy measures of early-life SES.^19^ However, the fact that a substantial IQ-mortality association persists beyond such adjustment is interesting, particularly given that the present study accounts for early-life SES in a different and more comprehensive way. Adjusting for within-family variance is notably more conservative than simply accounting for an explicit measure of SES, as it likely captures shared factors not directly related to SES such as genetic factors, environmental health (air pollution, etc.), childhood diet and exposure to passive smoking. Notably, adjusting for shared family factors led to a slightly larger attenuation of the intelligence–mortality association than adjusting for an explicit proxy measure of SES (see Supplementary Material, available as Supplementary Data at *IJE* online). However, the advantage of the family-based approach adopted in the present study is that it avoids any measurement error or residual confounding associated with using proxy measures such as parental occupation and income. Where previous studies have used proxy measures, residual confounding may erroneously underestimate the attenuating effect of early-life SES on the intelligence–mortality association.

As well as addressing the role of early-life SES in the intelligence–mortality association, the present study is the first to describe mortality risk in the siblings of the 6-Day Sample. The 6-Day Sample was formed to be representative of the Scottish nation born in 1936, and their younger siblings were followed up with the intention of examining life course outcomes within families. Notably, the present study replicated the significant association between IQ score and survival time previously reported in the 6-Day Sample,^24^ albeit with a much longer follow-up time (68 years). However, the present study is the first to demonstrate that the association between higher IQ scores and lower mortality risk extends to the younger siblings of the 6-Day Sample. Even in these younger individuals, individual differences in cognitive ability appear to have important implications for longevity.

### Limitations

By using family to represent SES, the present study only accounts for SES that is related to family circumstances. Individual factors related to early-life SES, such as birth weight,^19^ height^13^ and the person’s own education,^15^^,^^36^ may yet account for some of the association between intelligence and mortality. Previous work has suggested that adult SES may play a larger role in determining mortality risk than childhood SES, and that adjusting for adult SES and education provides the largest attenuation of the intelligence–mortality association.^10^ However, education and adult SES are phenotypically and genetically correlated with, and at least partly confounded by, childhood intelligence such that lower early-life cognitive ability may result in lower educational attainment, which leads to employment in riskier and generally lower-paid occupations.^37^^,^^38^

Although more conservative than adjusting for discrete proxy measures of SES, by adjusting for family-related factors, the present study assumes that the important aspects of the early-life environment are shared equally by siblings. In some cases, this assumption may not hold, e.g. where siblings live in different households (e.g. with a divorced parent or after migration) or where siblings live in the same household at very different points in time (e.g. with much later siblings). Indeed, the birth distance between 6-Day Sample members and their siblings was as much as 22 years in some exceptional cases. However, when the analyses were repeated using only siblings born within 7 years of their 6-Day Sample proband, the pattern of results was very similar. The effect of family, even in these individuals most likely (temporally) to share a family environment, only slightly attenuated the association between higher intelligence and reduced mortality risk.

The present study demonstrates that the association between early-life cognitive ability and mortality risk remains even after the confounding effect of early-life SES is accounted for. Notably, the present study takes the conservative approach of treating all variance shared within families as relevant to SES. Examining the role of intelligence within families allows adjustment of the intelligence–mortality association without the need to operationalize or measure SES, and so avoids the potential residual confounding associated with adjusting for proxy measures of early-life SES. The present study demonstrates that, whereas they are undoubtedly important for longevity, early-life family conditions are not sufficient to explain the intelligence–mortality association frequently reported in cognitive epidemiology research. Future work should focus on elucidating the mechanisms by which early-life cognitive ability is associated with longer life.

## Supplementary Data

Supplementary data are available at *IJE* online.

## Supplementary Material

Supplementary Data
