# Supplementary material for: Intelligence and all-cause mortality in the 6-Day Sample of the Scottish Mental Survey 1947 and their siblings: testing the contribution of family background
Source: Int J Epidemiol. 2017 Aug 21;47(1):89–96. doi: 10.1093/ije/dyx168 (PMC5837228; doi:10.1093/ije/dyx168)
Supplement: Supplementary Data [file dyx168_ije-2016-12-1484-file002.docx]

**Supplementary Material**

*Including single-child families*

In the analyses presented in the main text, single-child families were removed to ensure that adjusting for family-related factors accurately accounted for shared circumstances. As there is only one data point, within-family effects cannot accurately be estimated for single-child families. However, selecting only multiple-child families may provide a somewhat biased sample, and may limit the generalisability of any findings.

To address this, we fitted the stratified multivariable model presented in the main text (including fixed effects of standardised IQ score and sex and stratifying by family) to the full sample of 2784 individuals additionally including the individuals from single-child families (N = 460) and individuals whose siblings were removed due to missing data (N = 17).

The results were almost identical to those from the analyses presented in the main text using only multiple-child families. There was a significant reduction in hazard associated with a standard deviation advantage in IQ score, HR = 0.78, *p* = 0.002, 95% CI [0.67-0.92], and with being female, HR = 0.47, *p* < 0.001, 95% CI [0.38-0.58]. The association between intelligence and mortality does not appear to be limited to multiple-child families.

*Siblings born within 7 years*

Given that some siblings included in the analyses were born much later (Max = 22.36 years) than their 6-Day Sample probands, we conducted sensitivity analyses to ensure that adjusting for family captured variance that was shared between all family members. In particular, those individuals born closer in time to their 6-Day Sample proband were more likely to share home environments and family circumstances.

The survival analyses presented in the main text were repeated using only 6-Day Sample members and siblings born within 7 years (N = 1713, after removing individuals from families with no siblings born within 7 years). For this sample, we created a full multivariable Cox proportional hazards regression model, including fixed effects of standardised IQ scores and sex and a stratifying effect of family. Hazard ratios for standardised IQ scores and sex were adjusted for all other variables.

Similar to the analyses involving all siblings regardless of distance, the full multivariable model demonstrated a significant reduction in hazard associated with a standard deviation advantage in IQ score, HR = 0.77, *p* = 0.004, 95% CI [0.65, 0.92], and with being female, HR = 0.43, *p* < 0.001, 95% CI [0.34, 0.55]. Even when restricted to individuals born close in time, accounting for shared family factors does not completely attenuate the association between higher intelligence and reduced mortality risk.

*Using an explicit measure of early-life SES*

By way of comparison with the conservative analysis using family in the main text, we conducted analyses using an explicit measure of early-life SES – father’s occupational social class (FOSC). Father’s occupation for each individual was recorded as part of the sociological schedule. Occupations were coded into one of 5 social classes using the UK’s 1951 Classification of Occupations,^1^ and then reverse-coded such that higher classifications indicated more skilled occupations and higher social class. Note that, unlike the ‘family’ variable used in the main text, 6-Day Sample members and their siblings could have different observations of FOSC as measurements were obtained at different times.

As in the main text, survival analyses were conducted using Cox proportional hazards regression. In the first set of analyses, the fixed effect of either standardised IQ scores, sex, or family size were assessed including FOSC as a random effect. In the second set of analyses, these fixed effects were adjusted for all other predictors with FOSC included as a random effect. In the third set of analyses, the effects of standardised IQ scores, sex, and family size were assessed after adjusting for all other predictors and for a fixed effect of FOSC.

--- Insert Table 1 around here---

Taken alone, a one-level increase in FOSC was associated with a 15% reduction in mortality risk, HR = 0.85, 95% CI [0.79-0.92], *p* < 0.001. Table 1 shows the hazard ratios for standardised IQ scores, sex, and family size for each of the three stages of analysis. Notably, the significant association between higher standardised IQ scores and reduced mortality risk remained even after adjusting for FOSC, sex, and family size, with a 26% reduction in mortality risk with a single standard deviation increase in IQ score. This is similar to the analyses presented in the main text, in which the effect of standardised IQ score survived adjusting for shared family factors. Consistent with the analyses presented in the main text, being female was associated with a significant reduction (46%) in mortality risk, but a single member increase in family size was associated with only a small change in mortality risk.

As expected, including a stratifying effect of family proved a much more conservative method of accounting for early-life SES than adjusting for a discrete proxy measure of early-life SES. The hazard ratio associated with standardised IQ scores was higher when all shared family factors were taken into account (HR = 0.79) than when only FOSC was taken into account (HR = 0.74). It is unclear, however, what the source of this additional attenuation is other than that it relates to the shared family environment. This may indicate that accounting for all shared family factors more accurately captures shared early-life SES than a single discrete measure, or that it additionally captures factors beyond SES which are important for predicting longevity.

*References*

1. General Register Office. *Census 1951: Classification of occupations.* London, UK: Her Majesty's Stationery Office, 1956.

**Table 1.** Hazard ratios (HR) showing the mortality risk associated with a one standard deviation increase in IQ score, with being female, and with a one person increase in family size. Shown are the HRs including the random effect of father’s occupational social class (FOSC; in the univariable models), adjusted for the other predictors and the random effect of FOSC (in the multivariable model), and fully adjusted for all three predictors and the fixed effect of FOSC (in the fixed effects multivariable model; N = 2211)

|  | Univariable | | | Multivariable | | | Fixed Multivariable | | |
| --- | --- | --- | --- | --- | --- | --- | --- | --- | --- |
|  | HR | 95% CI | p | HR | 95% CI | p | HR | 95% CI | p |
| Standardised IQ score | 0.77 | 0.69-0.85 | <0.001 | 0.74 | 0.66-0.82 | <0.001 | 0.74 | 0.68-0.81 | <0.001 |
| Sex (Female) | 0.59 | 0.43-0.74 | <0.001 | 0.54 | 0.39-0.70 | <0.001 | 0.54 | 0.46-0.63 | <0.001 |
| Family size | 1.02 | 0.99-1.05 | 0.200 | 0.99 | 0.96-1.02 | 0.450 | 0.99 | 0.96-1.02 | 0.354 |
